# Interactions among Drosophila larvae before and during collision

**DOI:** 10.1038/srep31564

**Published:** 2016-08-11

**Authors:** Nils Otto, Benjamin Risse, Dimitri Berh, Jonas Bittern, Xiaoyi Jiang, Christian Klämbt

**Affiliations:** 1 Institute of Neuro- and Behavioral Biology, Westfälische Wilhelms-Universität Münster, Münster, Germany; 2 Department of Mathematics and Computer Science, Westfälische Wilhelms-Universität Münster, Münster, Germany

## Abstract

In populations of Drosophila larvae, both, an aggregation and a dispersal behavior can be observed. However, the mechanisms coordinating larval locomotion in respect to other animals, especially in close proximity and during/after physical contacts are currently only little understood. Here we test whether relevant information is perceived before or during larva-larva contacts, analyze its influence on behavior and ask whether larvae avoid or pursue collisions. Employing frustrated total internal reflection-based imaging (FIM) we first found that larvae visually detect other moving larvae in a narrow perceptive field and respond with characteristic escape reactions. To decipher larval locomotion not only before but also during the collision we utilized a two color FIM approach (FIM^2c^), which allowed to faithfully extract the posture and motion of colliding animals. We show that during collision, larval locomotion freezes and sensory information is sampled during a KISS phase (german: Kollisions Induziertes Stopp Syndrom or english: collision induced stop syndrome). Interestingly, larvae react differently to living, dead or artificial larvae, discriminate other Drosophila species and have an increased bending probability for a short period after the collision terminates. Thus, Drosophila larvae evolved means to specify behaviors in response to other larvae.

Most animals move to find their prey or their appropriate mating partners, to avoid competition for resources or to engage in cooperation. The success of this goal-oriented locomotion strongly relies on the surrounding objects and animals. For example, avoiding collisions in densely populated areas requires an appropriate perception of the surrounding and complex locomotion maneuvers. In many insect clades such as Drosophila, females lay a large number of eggs close to a food source^1^ and thus hatching larvae have to cope with other moving larvae and to compete for limited resources. Drosophila larvae are attracted to areas already explored by other larvae via a pheromone triggered signaling pathway^2^. Larvae of different species release different cocktails of attractive pheromones^2^. Thereby, behavioral changes are instructed to route them to distinct areas in common food sources. This increases the relative density of conspecifics. It has also been shown that larvae aggregate to perform cooperative digging which may increase the feeding efficacy on solid food^3^,^4^,^5^. Moreover, larvae of two distinct Drosophila species avoid to pupate close to larvae of other species but preferentially pupate in the neighborhood of their conspecifics^6^.

How Drosophila larvae perceive other animals and communicate with each other is currently only partially understood. There is evidence that Drosophila larvae are able to interact with other larvae via visual or gustatory cues. For example, larvae are visually attracted to distinct motion of tethered siblings^7^. Larval vision is mostly mediated by the larval eyes called Bolwig’s organs that are located in small pouches flanking the cephalopharyngeal skeleton. The Bolwig’s organ comprises 12 photoreceptor neurons, four of which express the blue sensitive Rhodopsin (Rh) 5a and eight express the green sensitive Rh6 8. During feeding, larvae show negative phototaxis, which is reversed when wandering larvae leave the food and navigate towards a dry pupariation site^9^,^10^. Owing to the position of the Bolwig’s organs in the anteriorly directed pouches of the head, a preferential sensitivity to frontal light can be determined^11^.

During larval locomotion, go phases are interrupted by reorientation phases characterized by reduced locomotion velocity and intensive head bending. During this phase the Bolwig’s organs probe local light information to determine the direction of the successive run. To navigate away from direct illumination requires temporal procession of this sensory input^12^. In addition to the visual system, pheromone mediated communication systems have been described that ensure species-specific recognition of larvae^2^ but olfactory preference of individual larvae is not modulated by surrounding larvae^13^. All present studies, however, did not consider the influence of sensory input on posture and locomotion during collision since segmenting and thus quantifying individual animals in these situations is not trivial.

Here, we asked whether Drosophila larvae have evolved means to change their locomotion behavior specifically in response to other larvae in dense populations. To study these aspects, automated tracking and analysis tools are required. In the last years several setups and algorithms have been established allowing high-throughput approaches, which unfortunately can only partially resolve colliding larvae^14^,^15^,^16^,^17^,^18^,^19^. To analyze what happens during and after a collision event, the identities and posture of the colliding animals must be traceable. A straightforward solution to this problem is to genetically mark one larva by green fluorescent protein (GFP) expression. We recently showed that frustrated total internal reflection (FTIR) provides an unprecedented high contrast view on crawling animals and developed the FTIR-based imaging (FIM) setup^14^. For a simultaneous analysis of multiple animals with different markers we developed FIM^2*c*^ (FIM two color^20^). This technology now for the first time allows resolving interacting animals during a collision event in a multi-target imaging approach for high throughput experiments. Employing FIM^2*c*^, we analyzed larval collision behavior. Colliding larvae show a stereotypic behavioral sequence upon contact to scan the touched object. During a one to two seconds lasting KISS phase (German: Kollisions Induziertes Stopp Syndrom, or in English: collision induced stop syndrome) larvae sample the specimen and behave differently when the collision object is made of unrelated material, a dead or a living larva of the same or a related Drosophila species. Our studies support the hypothesis that Drosophila larvae perceive the presence of other larvae and reveal a stereotypic influence on behavior which has to be considered carefully in multi-animal tracking approaches.

## Results

### Do larvae avoid or pursue collisions?

*D. melanogaster* larvae feed on moist substrates and leave the food during the third instar wandering stage. During their migratory path they often touch other animals. It was previously shown that larvae are able to use spatially and temporally coded visual stimuli to distinguish relatively complex visual patterns and for example respond to quivering larvae tethered above them^7^.

**Figure 1:**
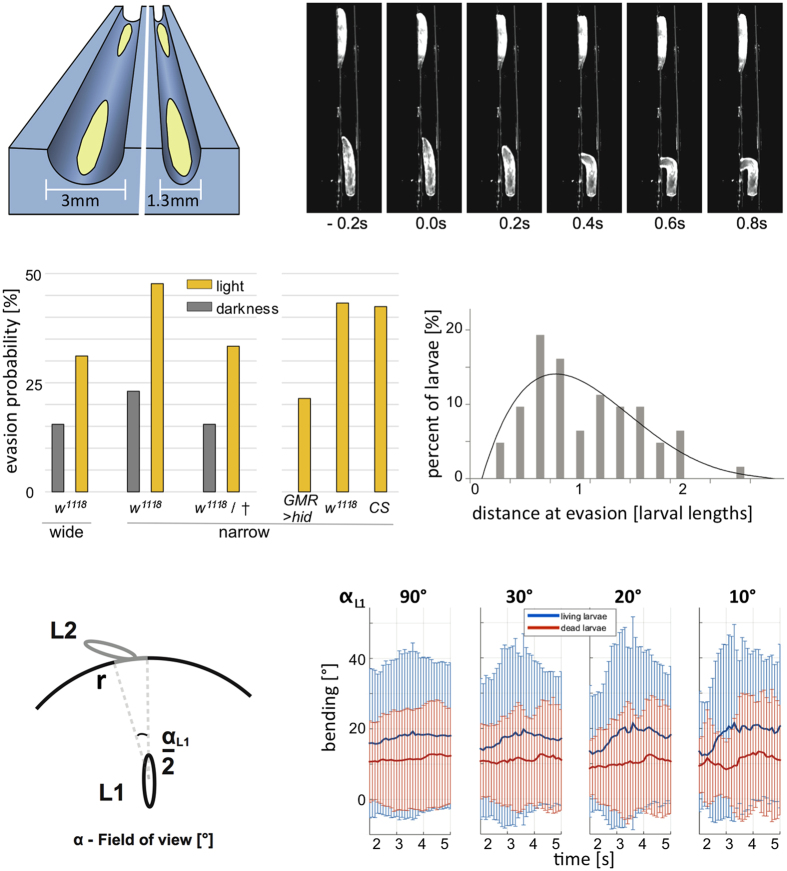
Larvae react to moving larvae in a narrow field of perception. (**a**) Schematic view of the SLIT assay. Larvae are placed opposing each other either in a wide (3 mm wide, 2 mm deep) or in a narrow (1.3 mm wide, 1.5 mm deep) trench. (**b**) Six still images of a movie showing an evasion reaction at about one larval length distance. (**c**) The evasion reaction depends on the lighting and the width of the trench. In the dark, only 15% of the larvae show evasion behavior in the wide trench, whereas 22% of the larvae show an evasion reaction in the narrow trench. Under normal light conditions 26% of the larvae show an evasion reaction in the wide trench and 47% of the larvae moving in the narrow trench evade. A similar evasion probability is noted when larvae crawl towards dead larvae in narrow trenches. When blind larvae (*GMR* *>* *hid*) are placed in the narrow trench 22.8% of them show an evasion reaction, whereas 42% of *Canton S* or 43.5% of *white*^*1118*^ (*w*^*1118*^) larvae show an evasion reaction. All reactions were tested to against crawling *da* *>* *GFP* larvae. (**d**) The distribution of distances at which the evasion reaction is initiated. The strongest reaction is initiated at a distance of about 0.8 larval length. (**e**) Schematic representation of the larval field of perception. The bending behavior of larva L1 is examined for 7 seconds upon entering of L2 in the field of view α of L1, provided that L2 stays within a circle with the radius *r*. (**f**) The bending probability in dependence of the field of view. Unconstrained larval movement was filmed with 10 frames per second for seven minutes. For each frame the mean bending angle and the standard deviation is indicated. The blue lines represent larvae (L1 in (**e**)) approaching a living larva (L2 in (**e**)), the red lines represent larvae (L1 in (**e**)) approaching dead larvae (L2 in (**e**)). Bending analysis was performed in dependence of the field of view as defined in (**e**). When the field of view is ≤20° an increase in body bending is observed only when approaching living larvae about 3–4 s after the larvae have entered the field of view. See Supplementary Fig. 1 for quantification.

Therefore, we expected that larvae would react to other larvae if close enough to be visually recognized. To test this, we established the SLIT (Single Larva Impact Trench) assay to analyze directed larval locomotion towards each other (Fig. 1a, Supplementary Movie 1). When placed into a trench (3 mm wide and 2 mm deep), 62% of the larvae moved straight forward in this trench (n = 37). If a second larva is placed in the same trench, but facing in opposing direction, the two animals move towards each other. When we placed larvae in the 3 mm wide trench, they can easily pass each other. In the dark we detected an evasion reaction only in 15% of the events (n = 39 events with two larvae of which in 6 cases one larva evaded and no case with both larvae showing an evasion reaction [n = 39/6/0]) (Fig. 1b,c). Interestingly, under normal light conditions in 31% of the cases at least one larva showed an evasion reaction (Fig. 1c, n = 119/37/3). This suggests that larvae are capable to use visual information to avoid collisions.

To further increase the likelihood for a collision we placed the animals in a narrow trench (1.3 mm wide and 1.5 mm deep) which does not allow the larvae to pass each other without touching. In the dark we noted an evasion reaction in 24% of the cases (n = 67/16/2) whereas 48% of the larvae showed this reaction given normal illumination (Fig. 1b,c; n = 65/31/8). When we repeated these experiments with larvae crawling towards dead larvae in the dark, we detected in 15% of the events an evasion reaction (Fig. 1c, n = 26/4/0) whereas in the light we noted in 33% of the cases an evasion reaction (n = 24/8/0). This supports the hypothesis that larvae are capable to perceive moving larvae visually. To further validate this finding we utilized larvae expressing the cell death gene *hid* in all photoreceptor cells, which renders the Bolwig’s organ blind (*GMR>hid*)^21^. These larvae were compared to *w*^*1118*^ larvae, which share the same genetic background as the *GMR>hid* larvae and to Canton S larvae. When placed into the narrow trench, only 22% of the blind larvae (n = 70) showed an evasion reaction, whereas 42% of the *Canton S* (n = 69) and 43% of *white*^*1118*^ larvae (n = 69) showed an evasion reaction (Fig. 1c).

The evasion reaction was strongest when the animals were about 0.8 larval-lengths apart (Fig. 1d). It is known that larvae are sensitive to temperature gradients^22^ but since Drosophila is poikilothermic we expect that larvae will not influence the temperature of the environment. Therefore, we anticipate that larvae recognize either vibration signals caused by the moving animal or recognize visual cues. Importantly, larvae appear to incorporate information regarding their environment (wide or narrow trench) to initiate an escape response or not. Moreover, these data show that larvae are able to avoid collisions specifically.

### Larvae have a narrow field of perception

In the SLIT assay evasion behavior is provoked using thin trenches. We next assayed larval collision behavior when many larvae moved in community and movements are not constrained by a prefigured trench. For this we monitored the simultaneous locomotion of 12 larvae on an agar arena (9.5 cm diameter) flanked by a salt barrier to confine larval movements. We first focused on non-colliding animals. We defined a field of view for a larva (labeled as L1 in Fig. 1e) with the azimuth of ± *α/2* and *r* = 10 mm (~2 larval lengths). An event is triggered for L1 if another larva enters the field of view (labeled as L2 in Fig. 1e). The bending behavior of L1 is then analyzed for several seconds to disclose a possible collision avoidance reaction. In a large field of view (*α* = 90°), almost no change in the mean bending probability can be detected (Fig. 1e,f, Supplementary Fig. 1). Upon narrowing of the field of view (*α* < 20°) there is a marked increase in the bending probability 4 seconds after the larvae entered the field of view (p = 0.002 comparing bending at t_2sec_ and t_4sec_). Given that the average locomotion speed of a third instar larva is about one larval length in 5 seconds this corresponds well to the preferred reaction distance we found in the SLIT assay (Fig. 1a–d). Interestingly, no significant increase in the bending rate is noted when larvae approach a dead larva (Fig. 1f, Supplementary Fig. 1) suggesting that larvae do not react to the shape of the larvae but rather react to the changing contrast of a moving larva.

### FIM^2*c*^ allows simultaneous detection of images of different wavelengths

**Figure 2:**
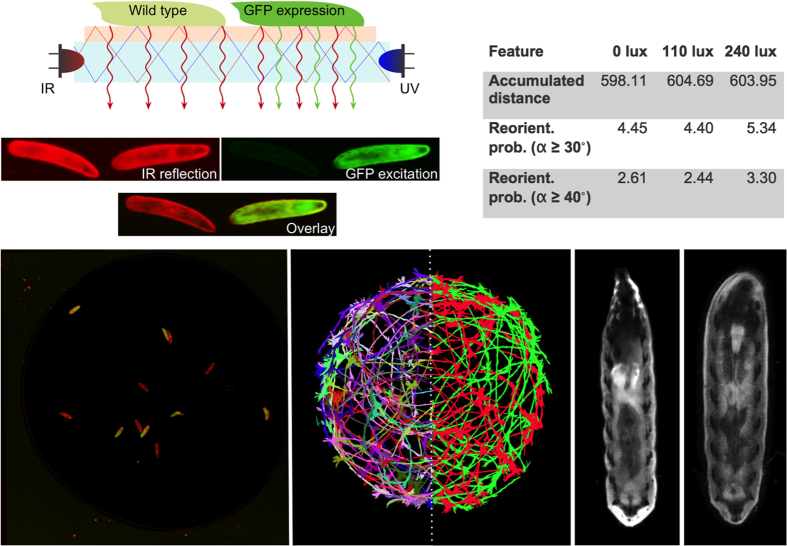
Imaging of GFP-expressing and non-expressing larvae using FIM^2c^. (**a**) The physical principles underlying FIM are independent of the wavelength used and work with infrared (IR) and ultraviolet light (UV). Reflected IR-light is captured by a camera equipped with an IR filter. UV-light excites GFP fluorescence which is detected using a second camera equipped with a UV-filter. Both views are merged into a two-color image. (**b**) Different intensities of UV-light do not significantly affect larval locomotion. (**c**) Image of a sample movie showing the detection of GFP-expressing animals (*da-Gal4* *>* *GFP*) and wild type animals. The two genotypes can clearly be separated. (**d**) Individual tracks are indicated by different colors. (**e**) Separation of GFP positive and GFP negative animals. (**f**) IR FIM^2c^ image of a third instar larva (Supplementary Movie 2). (**g**) UV FIM^2c^ image of a larva expressing GFP in the *nrv2* pattern. Note that the peripheral nerves can be seen (Supplementary Movie 3).

As pointed out above, larvae often collide but so far analysis of the collision event was not possible. To precisely study collisions we used a novel multi-color imaging system employing frustrated total internal reflection of infrared (IR) and ultraviolet (UV) light (FIM^2*c*^, Fig. 2)^14^,^15^,^20^. FIM^2*c*^ allows to simultaneously image distinct genotypes (e.g. GFP^+^ and GFP^−^), so that two colliding animals can be analyzed (Fig. 2c–e). Due to the physical principles underlying FIM^2*c*^, larvae are imaged at a high signal-to-noise ratio (Supplementary Movie 2). Even internal organs can be determined within these free crawling assays by expressing GFP in a tissue- or cell type-specific manner. For example, we used the *nrv2-Gal4* driver to express GFP in a small subset of glial cells. In the PNS, only three wrapping glial cells express *nrv2-Gal4* in every abdominal peripheral nerve^23^,^24^ which can be detected using FIM imaging highlighting the sensitivity of FIM^2*c*^ (Fig. 2g, Supplementary Movie 3). Last but not least using FIM^2*c*^ collisions can be resolved and the animals can be studied in unprecedented detail within high throughput assays.

### The influence of UV-irradiation on larval locomotion

To rule out possible side effects of UV-light on larval locomotion and thus possibly collision behavior, we first tested the effects of different UV-excitation strengths. Since the larval visual system is only composed of green- and blue- sensitive photoreceptor neurons (Rhodopsin 6 and Rhodopsin 5a), IR irradiation is not expected to affect behavior. Moreover, IR illumination does not affect the temperature on the tracking arena^14^. However, UV-LEDs emit light with a dominant wavelength of 470 nm, which might induce light avoidance behavior of Drosophila larvae^25^. In order to identify changes in larval locomotion, we determined aberrations in the run behavior by quantifying the accumulated distance after 90 seconds. In addition, the number of head sweeps was quantified to determine the probability for reorientation events^26^. We analyzed more than 100 animals each at different UV irradiations ranging from 0 to 240 lux and detected almost no change in the overall traveled distance (Fig. 2b). Similarly, the probability of reorientation events (bending angle ≥30° or ≥40°) did not change significantly. We only noted a slight increase in the number of reorientation events at higher UV irradiation levels (Fig. 2b). Whereas we measured almost no influence of constant UV-light on the locomotion of third instar larvae we did observe differences in larval reorientation during transitions from light on to light off or vice versa. In our experiments we subsequently used a constant lighting intensity of 100 lux. In conclusion, FIM^2*c*^ allows to image genetically distinct larvae with nearly no effects on locomotion behavior.

### Larval collision phases

Despite the ability of larvae to induce evasion reactions, we still observe collisions. This prompted us to study locomotion behavior during larval collisions and ask whether relevant information might be transmitted during contact. We placed six *white*^*1118*^ larvae together with six larvae ubiquitously expressing GFP on an agar arena (9.5 cm diameter with salt barrier, ubiquitous GFP expression is mediated by the following genotype: *daughterless-Gal4 (da-Gal4), UAS-CD8GFP*) and allowed the larvae to crawl freely in the absence of stimuli for 7 minutes. Imaging was done using FIM^2*c*^ (Fig. 2). Only collisions between single GFP-expressing and single non GFP-expressing animals with no overlays were counted as valid collisions. We extracted more than 1,400 resolved collisions in total. Larval collisions can be classified based on the duration of larval contacts and the duration of traceability before and after the collision. 25% of the collisions are very short and last less than 0.5 seconds whereas the other collisions last longer (Supplementary Fig. 2). Unless noted, we analyzed the behavior of the *white*^*1118*^ larvae involved in the collision and discarded the information relating to the GFP expressing animals.

**Figure 3:**
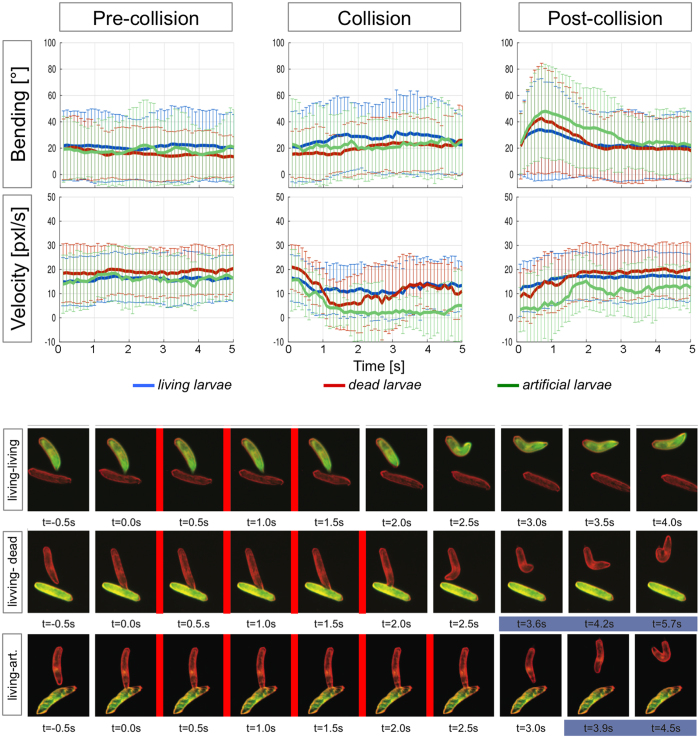
Collision behavior of third instar Drosophila larvae. (**a–f**) We extracted 358 valid collisions from our database when larval tracks were available one second before and one second after the collision. We plotted the median bending angle and its standard deviation (**a–c**) as well as the median velocity and the standard deviation (**d–f**). Data of larvae involved in collisions with GFP-expressing living larvae are indicated by blue lines, larvae involved in collisions with dead larvae are indicated by red lines and larvae involved in collisions with artificial larvae are indicated by green lines. (**a**,**d**) Before a collision, bending rate and velocity are constant. No differences were observed in larvae approaching a living, a dead or an artificial larva. (**b**,**e**) During collision, a characteristic KISS phase can be noted. Here velocity is reduced but the bending rate is unchanged. Upon collision with a living larva (blue lines), bending rate and velocity gradually increased during the collision. Upon collision with an artificial larva (green line), the KISS phase is dramatically increased in length. Upon collision with a dead larva (red line), intermediate values are found. (**c**,**f**) After collision, a short increase in the mean bending rate is observed, while the velocity gradually increases to normal levels. Interestingly strongest turning rates were noted upon collision with artificial larvae and intermediate turning rates were noted upon collision with dead larvae. (**g**) Stills of movies showing collisions between living Drosophila larvae, between living and dead larvae and living and artificial larvae. The red bars connect images showing the KISS phase. Time is indicated in seconds. Note, that the time scale is different at the end of the different video sequences (underlined in purple). See Supplementary Movies 4–6.

**Figure 4:**
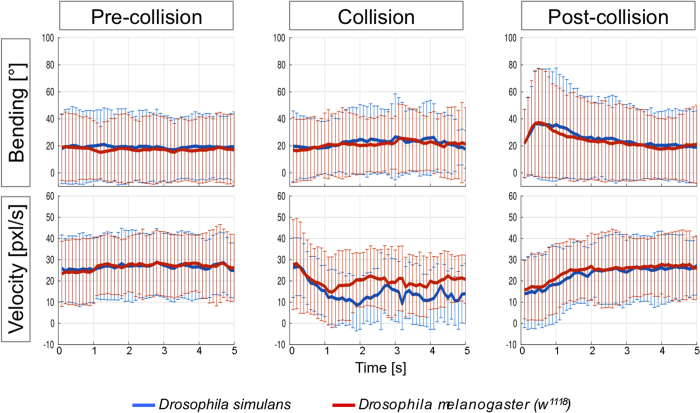
*Drosophila melanogaster* larvae react differently to *D. simulans* larvae. Collision analysis of GFP expressing *Drosophila melanogaster* larvae with either *Drosophila simulans* larvae or *D. melanogaster* larvae were analyzed as described in (**a,d**) Before collision, no differences in bending rate or velocity were noted. (**b**,**e**) During collision, GFP expressing *D. melanogaster* larvae discriminate between *D. melanogaster* larvae and *D. simulans* larvae. When colliding with the latter the sampling phase after the KISS phase is more pronounced. (**c**,**f**) After collision no differences in larval behavior were noted.

**Figure 5:**
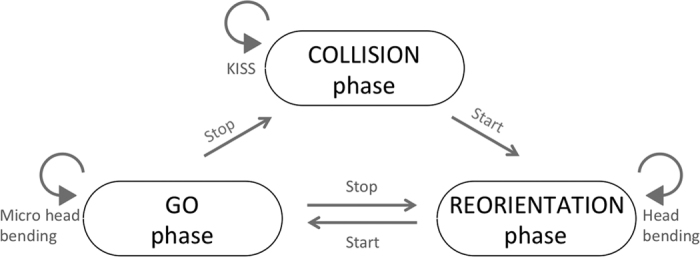
Schematic view of larval locomotion behavior. Larval locomotion can be separated into several distinct phases. During the go-phase only micro head bends are noted while the animal is moving about one larval length in 5 seconds. From the go-phase the animal can proceed to a collision- or to a reorientation-phase. The reorientation-phase is characterized by pronounced body bends and a reduction in locomotion velocity. From the reorientation-phase the animal can proceed to the go-phase. The collision-phase begins with a KISS characterized by reduced body bends and reduced velocity. The collision-phase is mostly ended by a reorientation-phase.

In the following we extracted 358 distinct collisions from the overall 1,400 collisions that lasted >0.5 seconds with animals traceable and not involved in any other collisions ≥1 seconds before and after the analyzed collision (Figs 3, 4, 5). The median collision length in this set is 2.5 seconds (Supplementary Fig. 3a). Three distinct phases of a collision can be defined: (1) a pre-collision phase, (2) a collision phase and (3) a post-collision phase.

In the pre-collision phase, velocity and bending are unchanged from normal undisturbed locomotion (Fig. 3a,d blue lines). In this respect it should be noted that all situations analyzed lead to collisions (i.e. larvae successfully avoided a collision are not included here).

In the collision phase we noted a characteristic time window during which locomotion speed dropped significantly (p < 0.00001, for details see supplementary Fig. 4) but no increase in body bending can be measured (Fig. 3b,e, blue lines, Supplementary Movie 4). We named this period KISS phase (German: Kollisions Induziertes Stopp Syndrom, or in English: collision induced stop syndrome). The KISS phase lasted usually 1–2 seconds after which larvae even when still engaged in a collision event resumed their normal locomotion speed. After the KISS phase a slight increase in bending probability could be noted (Fig. 3b,e blue lines).

In the post-collision phase, locomotion speed gradually increased and we noted a short and significant increase in the bending probability (Fig. 3c,f blue lines, p < 0.0001, for details see supplementary Fig. 4), which initiates the reorientation behavior after the termination of the collision.

### Larvae reveal different behaviors in the presence of different collision partners

The stereotypic reactions of larvae during as well as shortly after collisions indicate a significant change in behavior caused by the touch. To further investigate if this behavior changes due to different collision objects, we generated fluorescent artificial larvae (see Materials and Methods). In each trial, six of these artificial larvae were placed in the arena together with six unlabeled larvae (*w*^*1118*^) and their collision behavior was assayed. Before collision, bending probability and velocity remained constant as observed in collision of living Drosophila larvae (Fig. 3a,d, green line, n = 53 collision events). Interestingly, although collisions do not last longer, larval locomotion speed is still strongly reduced after the KISS phase (Fig. 3b,e, Supplementary Figs. 3a and 4, Supplementary Movie 5). The bending is again only slightly increased after the KISS phase during the collision event but dramatically increases in the beginning of the post-collision phase (Fig. 3c–f; p < 0.0001, see supplementary Fig. 4 for details). This change in behavior during and after the collision indicates that larvae sense a difference between artificial and living collision partners (statistical difference in velocity upon collision with living versus artificial larvae at t_1.5sec_ p = 2 × 10^−4^, at t_2.0sec_ p = 5 × 10^−8^). Most likely distinctive cues are sampled during the KISS phase and integrated into subsequent behavior.

To discriminate whether larvae use gustatory or haptic cues and to rule out the influence of locomotion, we confronted moving larvae with dead third instar larvae. We placed GFP expressing third instar larvae for 120 seconds in 60 °C hot air which is expected not to remove or disintegrate the long-chain fatty acid pheromones present on the larval cuticle^2^ and used the dead animals only for one recording. *w*^*1118*^ larvae colliding with dead larvae take longer to resume to their normal locomotion speed as compared to collisions with larvae that collide with living third instar larvae (Fig. 3b,e, difference living versus dead larvae at t_1.5sec_ and at t_2.0sec_ p = 2×10^−6^, see Supplementary Movie 6). Moreover, the length of collision time is increased (Supplementary Fig. 3a). In the beginning of the post-collision phase, the bending probability is in-between the measurement of animals colliding with artificial or living animals while the normal locomotion speed is resumed (Fig. 3e,f, red line, n = 175). These data demonstrate that larvae are able to discriminate their collision partner by gustatory and haptic cues independent of motility of the collision object. Again, this discrimination is likely to be sampled during the KISS period.

Previous results demonstrated that larvae can use chemical signals to communicate with their neighbors^2^,^6^,^27^. Third instar larvae of *Drosophila simulans*, a closely related fly species, do not generate the chemical cues that are attractive to *D. melanogaster*^2^. We therefore assayed collisions between *D. melanogaster* and *D. simulans*. In this paradigm, we tested GFP expressing *D. melanogaster* during the collision with non GFP expressing *D. simulans* larvae (n = 179) and compared this to collisions with living *w*^*1118*^ larvae (n = 278). Note that Gal4 expressing animals are generally a little faster due to slightly increased body size (compare Figs 3d and 4d). As for all other collision events, velocity and bending probability were constant before the collisions (Fig. 4a,d, see supplementary Fig. 4 for statistical analysis). The KISS period was slightly prolonged and little later initiated when comparing velocity during collision of *D. melanogaster* GFP-expressing larvae with *D. simulans* or *D. melanogaster* larvae (Fig. 4e; p_t1.5sec_ = 0.5; p_t2.0sec_ = 0.009). In addition, the median collision length is prolonged comparing *D. melanogaster* – *D. simulans* with *D. melanogaster* – *D. melanogaster* collisions (Supplementary Fig. 3b). Interestingly, when Gal4 expressing *D. melanogaster* were confronted with dead *w*^*1118*^ larvae (n = 165), collision length was much longer when compared to collision with dead *D. simulans* larvae (n = 76; p_t1.5sec_ = 0.018; p_t2.0sec_ = 0.002; Supplementary Figs 3c,4,5). This suggests that the discrimination between different species can also be achieved during the KISS phase. Physical contact with larvae of different species or with dead larva of the same species increases the duration of the collision period, indicating an overall less aversive stimulation.

### Three phases of larval locomotion when navigating in groups

Larval locomotion is usually classified into two distinct routines: After a phase of uninterrupted crawling the animals pause for self-induced reorientation^28^,^29^,^30^,^31^. FIM^2*c*^ now allows to add the obstacle-induced COLLISION phase as a novel component of larval locomotion. Unrestricted locomotion is the GO phase where regular peristalsis ensures forward movement (Fig. 5). Here only micro head bends can be seen. GO phases usually last for several seconds and are followed by a REORIENTATION phase characterized by stronger bending at a reduced velocity. The REORIENTATION phase triggers a new GO phase. However, a GO phase can also be terminated by a collision event. In case the object appears in a relatively narrow field of view the larvae use primarily visual cues to avoid collisions. This is in contrast to a report that larvae preferentially move below a group of tethered but still living larvae^7^. After a COLLISION phase a larva cannot directly switch to the GO phase but rather initiates a REORIENTATION phase. The COLLISION phase almost always comprises a KISS phase which differs from a typical REORIENTATION phase. In a KISS phase the velocity is reduced as well but no body bending can be measured suggesting a sampling of sensory information.

## Discussion

Whenever animals move in dense populations recognition of interaction partners and environment is beneficial. For example, Drosophila larvae show a cooperative burrowing behavior and prefer to pupate with conspecific larvae^1^,^2^,^3^,^4^,^5^. Here we studied the interaction of larvae with their surroundings before and during collisions to unravel the behavioral programs controlling community influences. For the latter analysis we employed a novel multi-target tracking system and found that larvae recognize other larvae in a narrow perceptive field already before collision.

Our data demonstrate that larvae can react upon detection of moving animals in a small field of perception and may avoid collisions in dependency of lighting and motility of the opposed object. In case a collision occurs, the opponent is sampled during a short KISS phase characterized by a “frozen” body posture. During this time living *D. melanogaster* larvae are distinguished from inanimate matter, dead larvae, or other species. The fact that *D. melanogaster* can discriminate *D. melanogaster* and *D. simulans* suggests that gustatory cues are utilized. Likewise gustatory cues are used to establish social networks in adult Drosophila^32^. Since *D. melanogaster* can also discriminate living and dead and even artificial opponents we anticipate that haptic cues are sampled as well. If neither larva-like sensory nor haptic cues are present (e.g. artificial matter), the KISS phase and thus the sampling period is extended.

In a recent report it was shown that larvae are attracted to moving objects in a light dependent manner^7^. Here we presented evidence that larvae rather tend to avoid other living animals provided they move in a narrow “field of view”. This corresponds to the structure the Bolwig’s organs embedded in anterior pouches in the head, which preferentially detect light coming from the front^11^. Since larvae react differently to living and dead larvae in the trench we anticipate that larvae integrate the changes of visual information over time rather than process complex images. In the SLIT assay we provoked collisions by forcing larvae to move onto each other in a precast trench. Surprisingly, larvae react different when moving in a narrow or a wide trench. This could indicate that the “field of view” is indeed very narrow and in the wide trench larvae do not see each other as often as in the narrow trench. Alternatively, larvae also integrate environmental features since pursuing a collision in a trench too small for two larvae to pass results in an increase of collision avoidance behavior. A related correlation of body size and the environment has been observed to modulate adult walking flies or stick insects that need to bridge a gap or in block climbing cockroaches^33^,^34^,^35^. Thus, knowledge on body size can be calculated by the insect brain and may already be present in third instar larvae.

To further test this hypothesis, advanced imaging technologies such as FIM^2c^ as introduced here are of great advantage. Although the resolution and the signal-to-noise ratio of most imaging setups is sufficient for tracking, the segmentation of individual animals shape fails during a collision event. In combination with sophisticated optogenetic or thermogenetic tools^36^,^37^,^38^,^39^,^40^ FIM^2c^ allows to use UV-light to modify neuronal activity in a temporally controlled manner while simultaneously recording larval behavior (data not shown). Higher sensitivity cameras may even allow the detection of GCaMP signals in groups of behaving animals.

The expression of fluorescent markers has been previously used to classify populations of adult Drosophila flies within the same experiment^41^. The Fluorescence Behavioral Imaging (FBI) tracker employs transgenic fluorescence and imaging hardware to discriminate courtship partner choice in genetically mixed groups of Drosophila but behavior during collisions cannot be addressed by this setup. The idTracker^19^ generates a characteristic optical fingerprint from each animal which allows to maintain identity before and after the collision. However, the method is not able to resolve the locomotion pattern during the collision event. We have now established a database with 1,400 fully resolved collisions and we envision that this ground truth will facilitate further mathematical modeling, including advanced representations like *Eigenworms* and Sequential Monte Carlo methods may lead to stable assignment of identity and posture resolution during collision events^18^,^42^,^43^.

In conclusion, we have demonstrated that Drosophila larvae react to others and can avoid collisions in a lighting dependent manner. However, larvae exhibit reactions only when moving animals appear in a narrow “field of view”. In case collisions occur we identified a stereotyped response pattern initiated by a KISS phase that eventually leads to the discrimination of living or dead animals and allows the detection of conspecific larvae. This suggests, that larvae sample their surroundings for information about other animals while navigating. Most likely this information is instructive for behavior also in the natural habitat. Considering the observed community effects, the population density has to be taken in account when performing larval locomotion assays. Moreover, the fact that larvae behave different in a wide or a narrow trench suggests that cues regarding larval environment feed into the execution of complex motor programs.

## Material and Methods

### Drosophila culture and strains

Drosophila larvae were raised on standard food at 25 °C in a 12 h dark-light cycle with 65% humidity. For imaging only third instar wandering stage larvae were collected 116 hours after egg laying. The following genotypes were used: *D*. *simulans*, *D*. *melanogaster white*^*1118*^, *Canton S*, *daughterless-Gal4* (*daGal4*) driving expression in all tissues, *nervana-Gal4* (*nrv2Gal4*) driving expression in the peripheral wrapping glial cells, GMR-Gal4 driving expression in all photoreceptor neurons, UAS-Hid (to drive expression of the proapoptotic gene *head involution defective* (*hid*). *UAS-CD8GFP* (to drive expression of a membrane tethered GFP). All fly strains were obtained from the Bloomington stock collection.

### General setup and preparations

Tracking experiments were performed on 0.8% agar in deionized H_2_O. For collision analysis the tracking surface is surrounded by a 2.5% agar barrier in 3M NaCl to keep animals confined to the tracking surface. All experiments were performed under room light conditions or in darkness. In the FIM2c setup infrared LEDs (HP HSDL-4230) and ultraviolet LEDs (LC503FBL1-15P-A3) were installed. The latter light sources have a dominant wavelength of 470 nm. For imaging a stereo-camera system is used (two Basler acA2040–25 gm cameras) with appropriate lenses (KOWA LM35HC). Besides the IR long pass filter (Schneider Kreuznach IF093), a custom GFP bandpass filter is mounted to the so-called GFP camera (AHF BrightLine HC 536/40). Recording and data analysis was carried out using a Fujitsu 520Celsius workstation. A new FIMTrack software module was created to match the requirements of FIM^2c^ (Supplementary Fig. 6; software available at: http://fim.uni-muenster.de). Note, that other and more than two colors can be integrated. Quantitative data visualization was done by custom-made MATLAB scripts. The non-parametrical Wilcoxon-Mann-Whitney-Test implemented in MATLAB was used as statistical test since a Gaussian distribution cannot be assumed.

### FIM^2c^ specific principles of illumination and recording

In the FIM^2c^ setup, light from both light sources enters the acrylic glass and is completely reflected at the glass / agar / air boundary as long as the angle of incidence is below the critical angle^14^,^15^,^20^. At the agar / larva transition light of both wavelengths enters the semi-translucent animal. The scattered infrared light is captured by an IR camera as described previously^14^. The UV light that enters the animals excites GFP which is detected by the GFP camera. This principle, as well as resultant images from both views, is given in Fig. 2a. Both cameras are monochrome cameras resulting in two monochrome detections, namely all animals in the IR image and GFP emissions in the GFP image. These measurements are merged into a RGB image whereas the red channel is used to store the IR image and the green channel is used to store the GFP image^20^.

### 2-Color collision resolution algorithm

The following conditions must be satisfied in order to resolve collisions within the FIM^2c^ system: Only two larvae participate within the collision, and one of the two animals must express GFP. In contrast to the algorithm described^14^, too big contours are not rejected and instead, the larvae participating in this collision are determined^20^. All larvae from time t − 1 having at least one point within the collision contour at time t are identified as participants. Collisions are assumed to be resolvable as long as only two larvae are involved in the collision and one of the two larvae is a GFP larva (b^GFP^ = true). The other larva has to be a non-GFP expressing animal (b^GFP^ = false). All collisions that are not satisfying both conditions are rejected. Resolvable collision contours are processed by first splitting the 2-color image into the red and green channel to segment the contour of the GFP expressing animal. All pixels within this collision contour are assumed to belong to the GFP larvae, all other pixels belong to the other animal. Afterwards, these contours can be used to calculate all features as described^14^,^20^. The result viewer tool from FIMTrack was used to validate the contour detection during collision resolution (>1,000 collisions).

### Generation of artificial larvae

We melted plastic hot sticks (Pattex PTK 56) and mixed it with fluorescein (Sigma). Threads of approximately 0.5 mm diameter were pulled out of the melted plastic and were cut into 5 mm long artificial larvae which after washing, were used in all experiments.

### Single larvae impact trench (SLIT) assay

To obtain trenches in the tracking arena we positioned thin and wide glass negatives of the desired trench into an agar slab casting tray. The slits were 5 cm long and 3 × 2 or 1.3 × 1.5 mm in diameter respectively. Several slabs were cut from casks each containing one trench and simultaneously imaged.

## Additional Information

**How to cite this article**: Otto, N. *et al*. Interactions among Drosophila larvae before and during collision. *Sci. Rep.* **6**, 31564; doi: 10.1038/srep31564 (2016).

## Supplementary Material

Supplementary Information

Supplementary video 1

Supplementary video 2

Supplementary video 3

Supplementary video 4

Supplementary video 5

Supplementary video 6

## References

[b1] Lin C.-C., Prokop-Prigge K. A., Preti G. & Potter C. J. Food odors trigger Drosophila males to deposit a pheromone that guides aggregation and female oviposition decisions. eLife Sciences 4, (2015). 10.7554/eLife.08688 PMC4621432 26422512

[b2] Mast J. D., De Moraes C. M., Alborn H. T., Lavis L. D. & Stern D. L. Evolved differences in larval social behavior mediated by novel pheromones. eLife Sciences 3, e04205 (2014). 10.7554/eLife.04205 PMC4270068 25497433

[b3] Durisko Z., Kemp R., Mubasher R. & Dukas R. Dynamics of social behavior in fruit fly larvae. Plos One 9, e95495 (2014). 24740198 10.1371/journal.pone.0095495 PMC3989340

[b4] Wu Q. . Developmental control of foraging and social behavior by the Drosophila neuropeptide Y-like system. Neuron 39, 147–161 (2003). 12848939 10.1016/s0896-6273(03)00396-9

[b5] Xu J., Sornborger A. T., Lee J. K. & Shen P. Drosophila TRPA channel modulates sugar-stimulated neural excitation, avoidance and social response. Nat Neurosci. 11, 676–682 (2008). 18469811 10.1038/nn.2119

[b6] Beltramí M., Medina-Muñoz M. C., Del Pino F., Ferveur J.-F. & Godoy-Herrera R. Chemical cues influence pupation behavior of Drosophila simulans and Drosophila buzzatii in nature and in the laboratory. Plos One 7, e39393 (2012). 22737236 10.1371/journal.pone.0039393 PMC3380841

[b7] Justice E. D., Macedonia N. J., Hamilton C. & Condron B. The simple fly larval visual system can process complex images. Nat Commun. 3, 1156 (2012). 23093193 10.1038/ncomms2174

[b8] Sprecher S. G., Pichaud F. & Desplan C. Adult and larval photoreceptors use different mechanisms to specify the same Rhodopsin fates. Genes & Development 21, 2182–2195 (2007). 17785526 10.1101/gad.1565407 PMC1950857

[b9] Godoy-Herrera R., Santander R. & Figueroa J. A developmental and biometrical analysis of larval photoresponse of Drosophila. Animal Behaviour 48, 251–262 (1994).

[b10] Sawin E., Harris L., Campos A. & Sokolowski M. Sensorimotor transformation from light reception to phototactic behavior in Drosophila larvae (Diptera: Drosophilidae). Journal of Insect Behavior. 7, 553–567 (1994).

[b11] Hinnemann A., Niederegger S., Hanslik U., Heinzel H.-G. & Spiess R. See the light: electrophysiological characterization of the Bolwig organ’s light response of Calliphora vicina 3rd instar larvae. Journal of Insect Physiology 56, 1651–1658 (2010). 20603127 10.1016/j.jinsphys.2010.06.010

[b12] Kane E. A. . (2013). Sensorimotor structure of Drosophila larva phototaxis. Proceedings of the National Academy of Sciences 110, E3868–77 (2013). 10.1073/pnas.1215295110 PMC3791751 24043822

[b13] Niewalda T., Jeske I., Michels B. & Gerber B. ‘Peer pressure’ in larval Drosophila? Biol Open 6, 575–582 (2014). 24907371 10.1242/bio.20148458 PMC4154293

[b14] Risse B. . FIM, a Novel FTIR-Based Imaging Method for High Throughput Locomotion Analysis. Plos One 8, e53963 (2013). 23349775 10.1371/journal.pone.0053963 PMC3549958

[b15] Risse B., Otto N., Berh D., Jiang X. & Klämbt C. FIM Imaging and FIMtrack: Two New Tools Allowing High-throughput and Cost Effective Locomotion Analysis. J Vis Exp. 24 (2014). 10.3791/52207 PMC4354465 25591081

[b16] Gershow M. . Controlling airborne cues to study small animal navigation. Nat Meth. 9, 290–296 (2012). 10.1038/nmeth.1853 PMC3513333 22245808

[b17] Swierczek N. A., Giles A. C., Rankin C. H. & Kerr R. A. High-throughput behavioral analysis in C. elegans. Nat Meth. 8, 592–598 (2011). 10.1038/nmeth.1625 PMC3128206 21642964

[b18] Fiaschi L. . Tracking indistinguishable translucent objects over time using weakly supervised structured learning. *Proceedings of IEEE Conf. on Computer Vision and Pattern recognition* 2736–2743 (2014).

[b19] Pérez-Escudero A., Vicente-Page J., Hinz R. C., Arganda S. & de Polavieja G. G. idTracker: tracking individuals in a group by automatic identification of unmarked animals. Nat Meth. 11, 743–748 (2014). 10.1038/nmeth.2994 24880877

[b20] Risse B. . FIM^2c^: A Multi-Colour, Multi-Purpose Imaging System to Manipulate and Analyse Animal Behaviour. *IEEE Trans Biomed Eng*. In press (2016).

[b21] Xiang Y. . Light-avoidance-mediating photoreceptors tile the Drosophila larval body wall. Nature , 468, 921–926 (2010). 21068723 10.1038/nature09576 PMC3026603

[b22] Klein M. . Sensory determinants of behavioral dynamics in Drosophila thermotaxis. Proceedings of the National Academy of Sciences 112, E220–E229 (2015). 10.1073/pnas.1416212112 PMC4299240 25550513

[b23] Hilchen von C. M., Bustos Á. E., Giangrande A., Technau G. M. & Altenhein B. Predetermined embryonic glial cells form the distinct glial sheaths of the Drosophila peripheral nervous system. Development 140, 3657–3668 (2013). 23903191 10.1242/dev.093245 PMC3915570

[b24] Matzat T. . Axonal wrapping in the Drosophila PNS is controlled by glia-derived neuregulin homolog Vein. Development 142, 1336–1345 (2015). 25758464 10.1242/dev.116616

[b25] Keene A. C. & Sprecher S. G. Seeing the light: photobehavior in fruit fly larvae. Trends Neurosci 35, 104–110 (2012). 22222349 10.1016/j.tins.2011.11.003

[b26] Lahiri S. . Two alternating motor programs drive navigation in Drosophila larva. Plos One 6, e23180 (2011). 21858019 10.1371/journal.pone.0023180 PMC3156121

[b27] Durisko Z. & Dukas R. Attraction to and learning from social cues in fruitfly larvae. Proceedings of the Royal Society of London B: Biological Sciences 280, 20131398 (2013). 10.1098/rspb.2013.1398 PMC3735258 23902906

[b28] Gomez-Marin A., Stephens G. J. & Louis M. Active sampling and decision making in Drosophila chemotaxis. Nat Commun. 2, 441 (2011). 21863008 10.1038/ncomms1455 PMC3265367

[b29] Gomez-Marin A. & Louis M. Active sensation during orientation behavior in the Drosophila larva: more sense than luck. Curr Opin Neurobiol. 22, 208–215 (2012). 22169055 10.1016/j.conb.2011.11.008

[b30] Berni J., Pulver S. R., Griffith L. C. & Bate M. Autonomous Circuitry for Substrate Exploration in Freely Moving Drosophila Larvae. Current Biology 22, 1861–1870 (2012). 22940472 10.1016/j.cub.2012.07.048 PMC4082562

[b31] Berni J. Genetic dissection of a regionally differentiated network for exploratory behavior in Drosophila larvae. Curr Biol. 25, 1319–1326 (2015). 25959962 10.1016/j.cub.2015.03.023 PMC4446794

[b32] Schneider J., Dickinson M. H. & Levine J. D. Social structures depend on innate determinants and chemosensory processing in Drosophila. Proceedings of the National Academy of Sciences 109, 17174–17179 (2012). 10.1073/pnas.1121252109 PMC3477376 22802679

[b33] Bläsing B. & Cruse H. Mechanisms of stick insect locomotion in a gap-crossing paradigm. *J. Comp. Physiol. A Neuroethol. Sens. Neural. Behav. Physiol*. 190, 173–183 (2004). 14735308 10.1007/s00359-003-0482-3

[b34] Pick S. & Strauss R. Goal-driven behavioral adaptations in gap-climbing Drosophila. Curr Biol 15, 1473–1478 (2005). 16111941 10.1016/j.cub.2005.07.022

[b35] Harley C. M., English B. A. & Ritzmann R. E. Characterization of obstacle negotiation behaviors in the cockroach, Blaberus discoidalis. J Exp Biol. 212, 1463–1476 (2009). 19411540 10.1242/jeb.028381

[b36] Owald D., Lin S. & Waddell S. Light, heat, action: neural control of fruit fly behaviour. Philosophical Transactions of the Royal Society B: Biological Sciences 370, 20140211 (2015). 10.1098/rstb.2014.0211 PMC4528823 26240426

[b37] Harris R. M., Pfeiffer B. D., Rubin G. M. & Truman J. W. Neuron hemilineages provide the functional ground plan for the Drosophila ventral nervous system. eLife Sciences 4, (2015). 10.7554/eLife.04493 PMC4525104 26193122

[b38] Tastekin I. . Role of the subesophageal zone in sensorimotor control of orientation in Drosophila larva. Curr Biol. 25, 1448–1460 (2015). 25959970 10.1016/j.cub.2015.04.016

[b39] Kohsaka H., Takasu E., Morimoto T. & Nose A. A group of segmental premotor interneurons regulates the speed of axial locomotion in Drosophila larvae. Curr Biol. 24, 2632–2642 (2014). 25438948 10.1016/j.cub.2014.09.026

[b40] Hernandez-Nunez L. . Reverse-correlation analysis of navigation dynamics in Drosophila larva using optogenetics. eLife Sciences 4, e06225 (2015). 10.7554/eLife.06225 PMC4466337 25942453

[b41] Ramdya P., Schaffter T., Floreano D. & Benton R. Fluorescence behavioral imaging (FBI) tracks identity in heterogeneous groups of Drosophila. Plos One 7, e48381 (2012). 23144871 10.1371/journal.pone.0048381 PMC3492344

[b42] Brown A. E. X., Yemini E. I., Grundy L. J., Jucikas T. & Schafer W. R. A dictionary of behavioral motifs reveals clusters of genes affecting Caenorhabditis elegans locomotion. Proceedings of the National Academy of Sciences 110, 791–796 (2013). 10.1073/pnas.1211447110 PMC3545781 23267063

[b43] Stephens G. J., Johnson-Kerner B., Bialek W. & Ryu W. S. Dimensionality and dynamics in the behavior of C. elegans. PLoS Comp Biol. 4, e1000028 (2008). 10.1371/journal.pcbi.1000028 PMC2276863 18389066

