# Supplementary material for: Interactions among Drosophila larvae before and during collision
Source: Sci Rep. 2016 Aug 11;6:31564. doi: 10.1038/srep31564 (PMC4980675; doi:10.1038/srep31564)
Supplement: Supplementary Information [file srep31564-s1.pdf]

Supplementary information

Interactions among Drosophila larvae before and during collision

Nils Otto*[1], Benjamin Risse*[1,2,3], Dimitri Berh[1,2], Jonas Bittern[1], Xiaoyi Jiang[2] and Christian Klämbt[1]

Supplementary Fig. 1 Larvae have a narrow field of perception

(**a**) The bending behavior of larvae approaching but not colliding with living or dead larvae was analyzed according to Fig. 1e. Two five frames long time intervals (t_1 and t_2) were defined 2s and 3.5s after the larva L2 entered the field of perception of larva L2 (red boxes). (**b**) Body bending changes plotted against the opening angle of the field of perception. The value was calculated as the quotient of the mean bending rate at t_1 and t_2. Larvae react to living larvae appearing in a narrow field of perception. The following numbers of larvae were included into the calculation for a field of perception of 90°. 474 larvae approaching living larvae and 305 larvae approaching dead larvae were included (90°: 474|305; 80°: 428|263; 70°: 381|220; 60°: 338|196; 50°: 291|154; 40°: 233|132; 30°: 174|113; 20°: 118|79; 10°: 65|50.

Supplementary Fig. 2 Larval collision examples

(**a-d**) Examples of collisions of third instar larva as resolved by FIM[2c]. (**e**) Logarithmic distribution of collision length. No preferred collision length is noted but rather collisions appear terminated with a constant probability.

Supplementary Fig. 3 Length of collision phase

Box plots showing the collision times (whiskers: 1.5x interquartile distance). The median is noted. Statistical significance is tested by a heteroscedastic two tailed students t-test; (*<,= 0.05; *** <= 0.005; ****<= 0.0005 (**a**) Collision length of non-GFP expressing larvae with living, dead or artificial larvae. Collisions with dead larvae resulted in the longest collision time. (**b**) The collision length is increased when comparing GFP-expressing *D. melanogaster* larvae colliding with *D. simulans* larvae with *D. melanogaster* (w^{1118}). (**c**) When GFP-expressing larvae collided with dead *D. melanogaster* (w^{1118}) or dead *D. simulans* larvae a marked difference in the collision phase length was noted.

Supplementary Fig. 4 Statistical analysis of collision and post-collision behavior

The p-values for comparing velocity at the last time point (5sec) before collision with all values during collision or comparing bending probability at the last time point before collision with all values after collision are shown using the data of Figures 3,4 and supplementary Figure 5 as indicated. The drop in significance observed towards the end of the analysis window (5 sec) is explained by the reduced numbers of long lasting collisions. The different genotypes are indicated.

Supplementary Fig. 5 Collision behavior with dead *Drosophila* larvae of different species

Collision analysis of GFP expressing *Drosophila melanogaster* larvae with dead *Drosophila simulans* larvae or dead *D. melanogaster* larvae (w^{1118}). For details see Fig. 3. (**a,d**) Before collision no differences in bending rate or velocity were noted over time. Likewise no major changes in bending rate and velocity were noted in the collision phase (**b,e**) or after collision (**c,f**).

Supplementary Fig. 6 The FIM^{2c} software features

Schematic description of features presented in this work now included in FIMtrack (NN = Nearest Neighbor; i.e. closest larva). The domain of the features can be binary (i.e. true or false), in degree (i.e. [0°, 180°]), natural numbers (i.e. **N**) or positive real numbers (i.e. $\mathbf{R}_+$).

Supplementary movie 1

SLIT assay. Two larvae are approaching each other in a narrow trench under normal lighting conditions (350 lux). The movie is taken with 10 frames/s at a resolution of 150 pixel per larval length.

Supplementary movie 2

Wild type (*w*^{1118}) third instar larvae imaged with infrared light. The movie is taken with 20 frames/s frame rate at a resolution of 380 pixel per larval length.

Supplementary movie 3

nrv2Gal4; UASGFP third instar larvae imaged with UV-light. The movie is taken with 20 frames/s at a resolution of 380 pixel per larval length. Asterisk denotes segmental abdominal nerves, the arrow highlights an individual *nrv2Gal* positive glial cell. The plus indicates the position of the ventral nerve cord.

Supplementary movie 4

Collision of a GFP expressing animal with a non-GFP expressing animal. Note the KISS phase at the beginning of collision. No movement is detectable. The movie is taken with 10 frames/s frame rate at a resolution of 100 pixel per larval length.

Supplementary movie 5

Collision of a non-GFP expressing animal with an artificial larva. Note the KISS phase at the beginning of collision. No movement is detectable. The movie is taken with 10 frames/s frame rate at a resolution of 100 pixel per larval length.

Supplementary movie 6

Collision of a non GFP expressing animal with a dead GFP expressing animal. Note the KISS phase at the beginning of collision. No movement is detectable. The movie is taken with 10 frames/s frame rate at a resolution of 100 pixel per larval length.

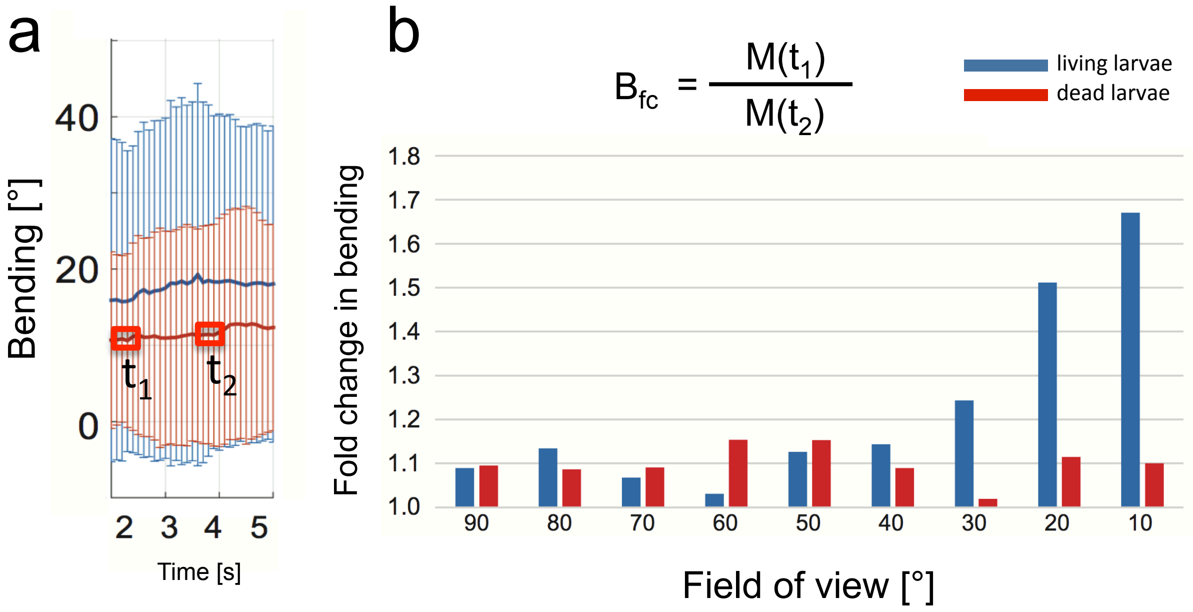
a
40
20
0
Bending [°]
t1
t2
2 3 4 5
Time [s]
b
Bfc = M(t1) / M(t2)
living larvae
dead larvae
1.8
1.7
1.6
1.5
1.4
1.3
1.2
1.1
1.0
Fold change in bending
90 80 70 60 50 40 30 20 10
Field of view [°]

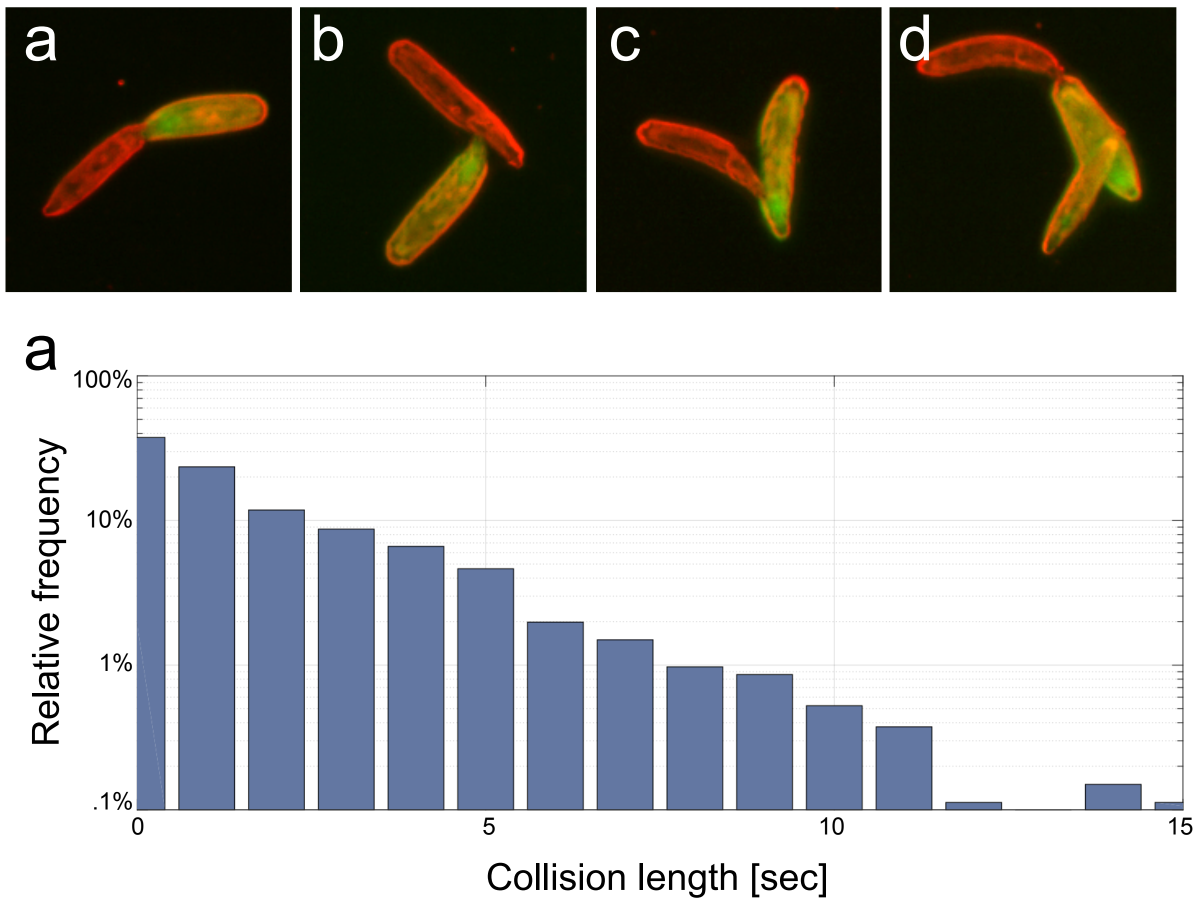
a
b
c
d
a
100%
10%
1%
.1%
Relative frequency
0
5
10
15
Collision length [sec]

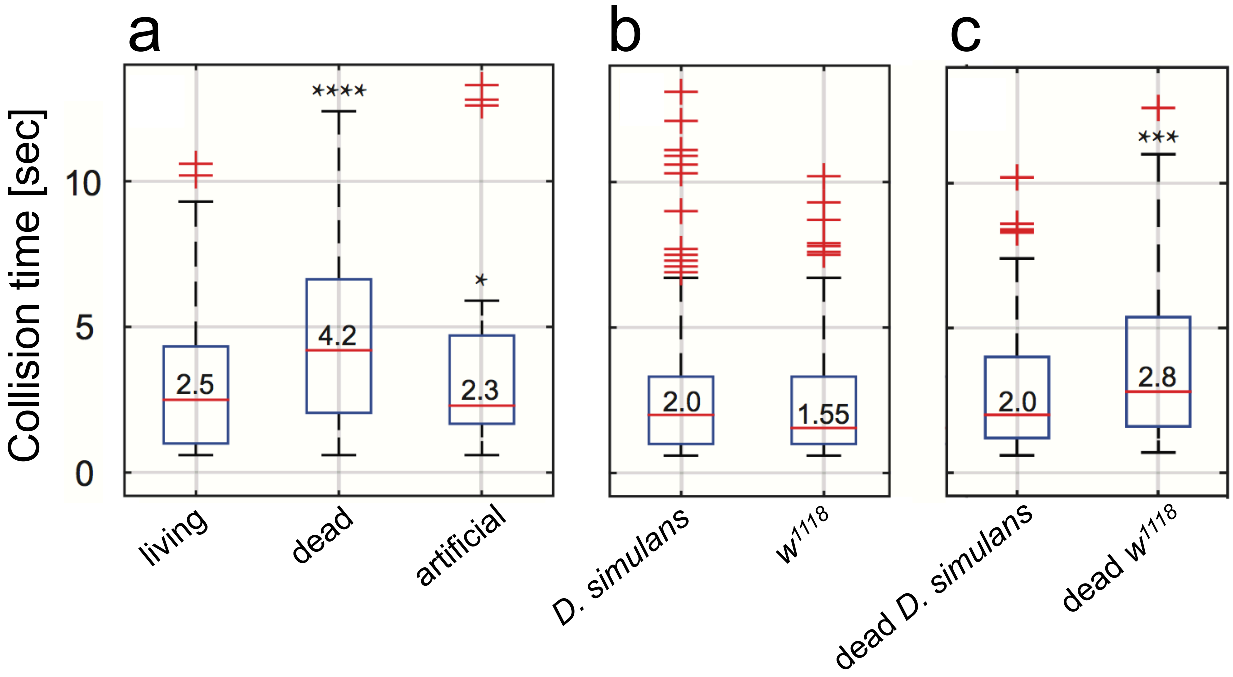
a
b
c
Collision time [sec]
0
5
10
2.5
4.2
2.3
****
*
2.0
1.55
2.0
2.8
***
living
dead
artificial
D. simulans
w1118
dead D. simulans
dead w1118

Otto et al., Supplementary Figure 4

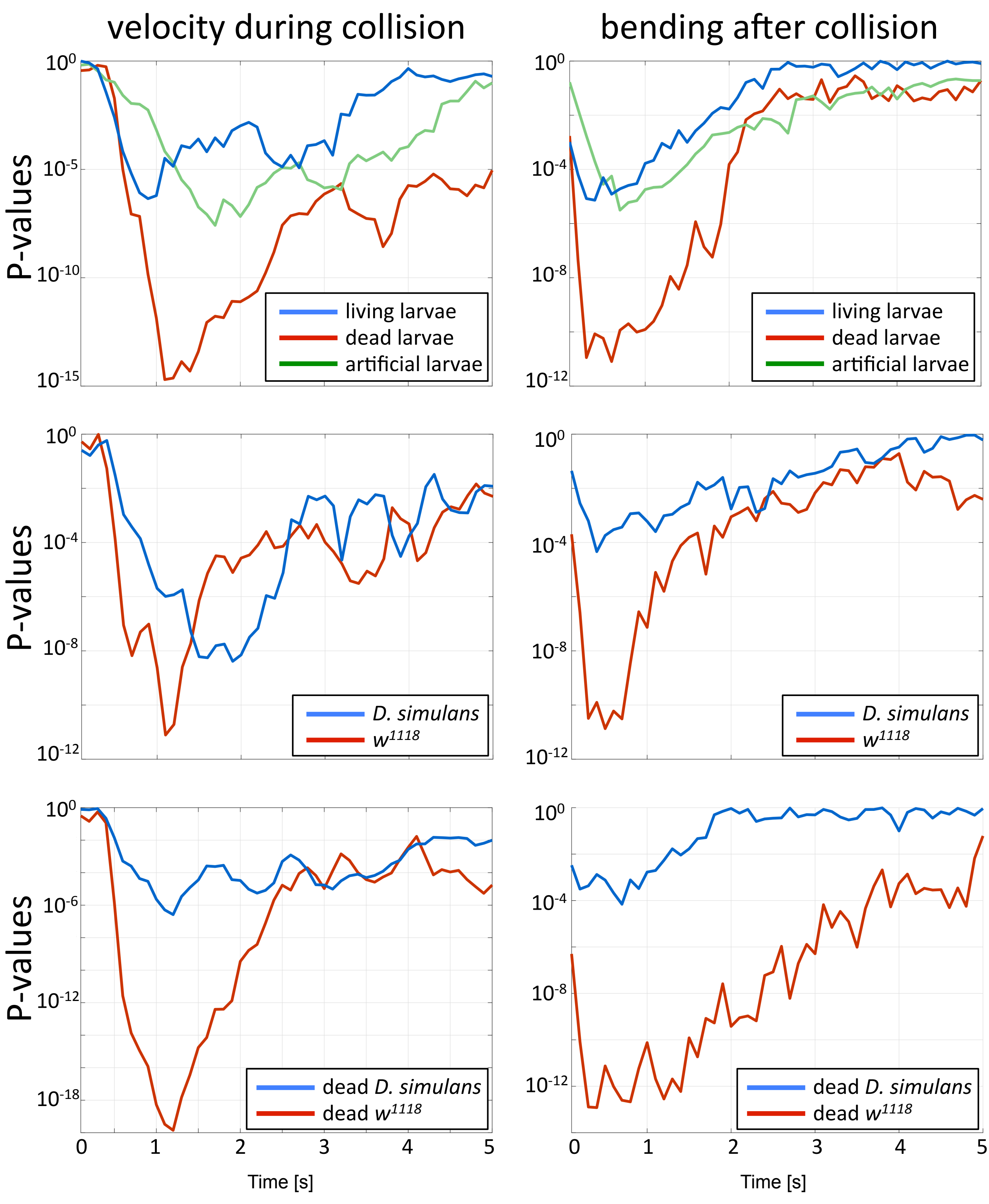

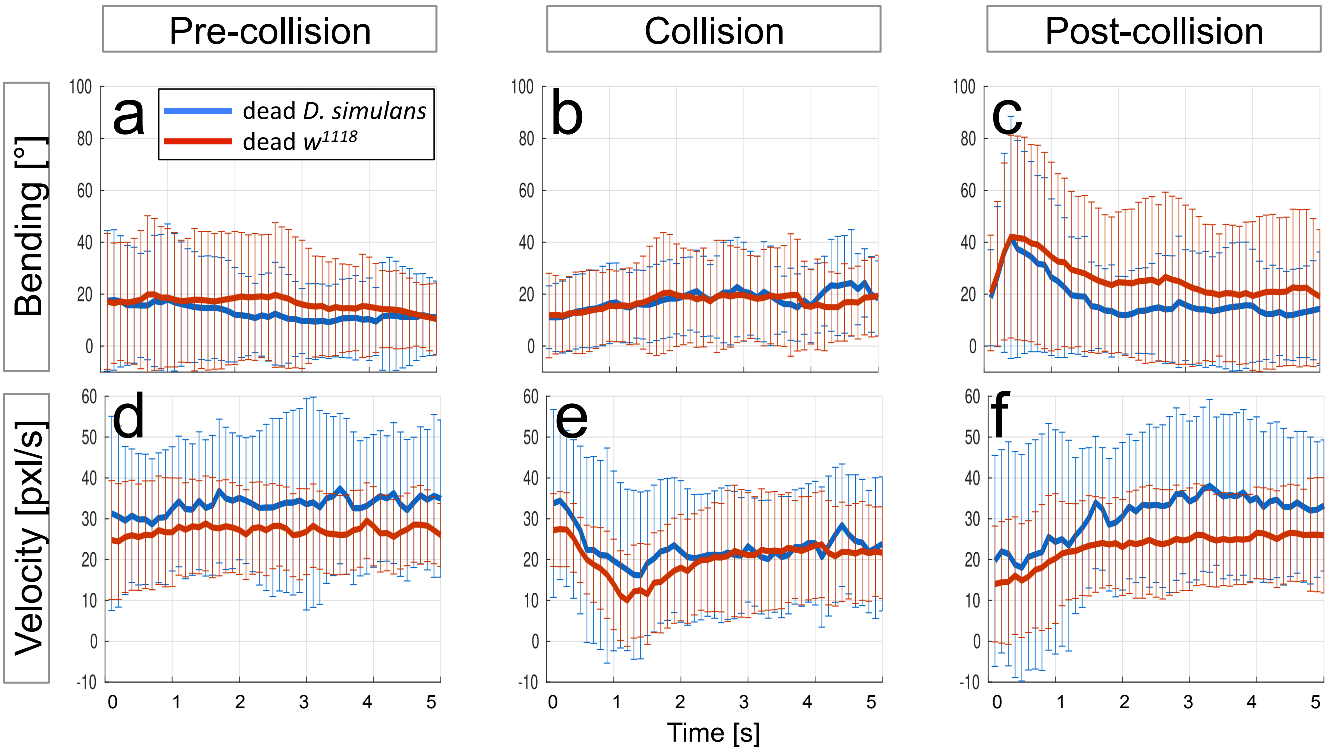
Pre-collision
Collision
Post-collision
Bending [°]
Velocity [pxl/s]
a
b
c
d
e
f
dead D. simulans
dead w1118
Time [s]

Feature	Sketch	Domain
Is GFP Larva	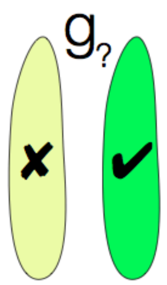	$g_? \in \{\text{true, false}\}$
Is in Collision	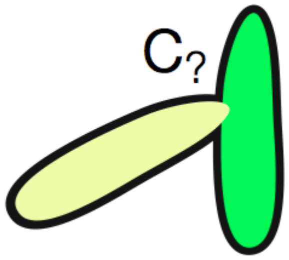	$c_? \in \{\text{true, false}\}$
Distance to NN	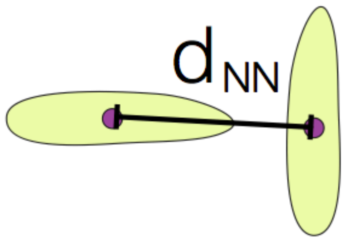	$d_{NN} \in \mathbf{R}_+$
Bearing to NN	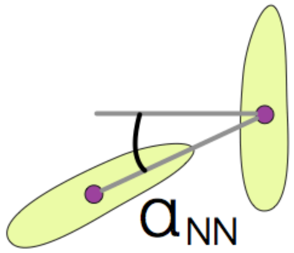	$\alpha_{NN} \in [0°,180°]$
Collision Length	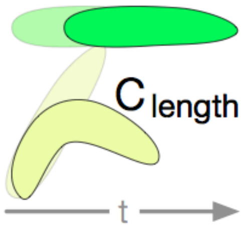	$C_{length} \in \mathbf{N}$
